# Left ventricular mechanical dyssynchrony by cardiac magnetic resonance is greater in patients with strict vs. conventional ECG criteria for left bundle branch block

**DOI:** 10.1186/1532-429X-15-S1-P152

**Published:** 2013-01-30

**Authors:** Linus Andersson, Katherine Wu, Bjorn Wieslander, Zak Loring, Terry Frank, Charles Maynard, Gary Gerstenblith, Gordon F Tomaselli, Robert G Weiss, Galen S Wagner, Martin Ugander, David G Strauss

**Affiliations:** 1 Cardiac MR group, Department of Clinical Physiology, Karolinska Institutet, Stockholm, Sweden; 2 Duke Clinical Research Institute, Duke University, Durham, NC, USA; 3 Department of Medicine, Johns Hopkins Medical Institutions, Baltimore, MD, USA; 4 Duke University School of Medicine, Duke University, Durham, NC, USA; 5 Department of Health Services, University of Washington, Seattle, WA, USA; 6 Office of Science and Engineering Laboratories, Center for Devices and Radiological Health, United States Food and Drug Administration, Silver Spring, MD, USA

## Background

Left bundle branch block (LBBB) is a marker of increased delay between septal and left ventricular (LV) lateral wall electrical activation, and is a predictor of which patients will benefit from cardiac resynchronization therapy (CRT). Recent analysis has suggested that one third of patients meeting conventional ECG criteria for LBBB are misdiagnosed and new strict LBBB criteria have been proposed. We tested the hypothesis that strict LBBB patients have greater LV mechanical dyssynchrony than patients only meeting conventional LBBB criteria, while there is no difference between patients with conventional-only LBBB and LV conduction delay with QRS duration 110-119 ms.

## Methods

Sixty-four cardiomyopathy patients referred for a primary prevention implantable cardioverter defibrillator (ICD) underwent 12-lead ECG and cardiac magnetic resonance (CMR) myocardial tagging. The patients were classified as strict LBBB, conventional-only LBBB or non-LBBB, indicating nonspecific LV conduction delay with QRS duration 110-119 ms. The time delay between septal and lateral LV wall peak circumferential strain, septal-to-lateral wall delay, was measured by CMR.

## Results

Patients with strict LBBB (n=31) had a greater septal-to-lateral wall delay, compared to patients with conventional-only LBBB (n=19) (210±137 ms vs. 122±102 ms, p=0.01). There was no significant difference between conventional-only LBBB and non-LBBB (n=14) septal-to-lateral wall delay (122±102 ms vs. 100±86 ms, p=0.51). The results are presented in the figure below.

**Figure 1:**
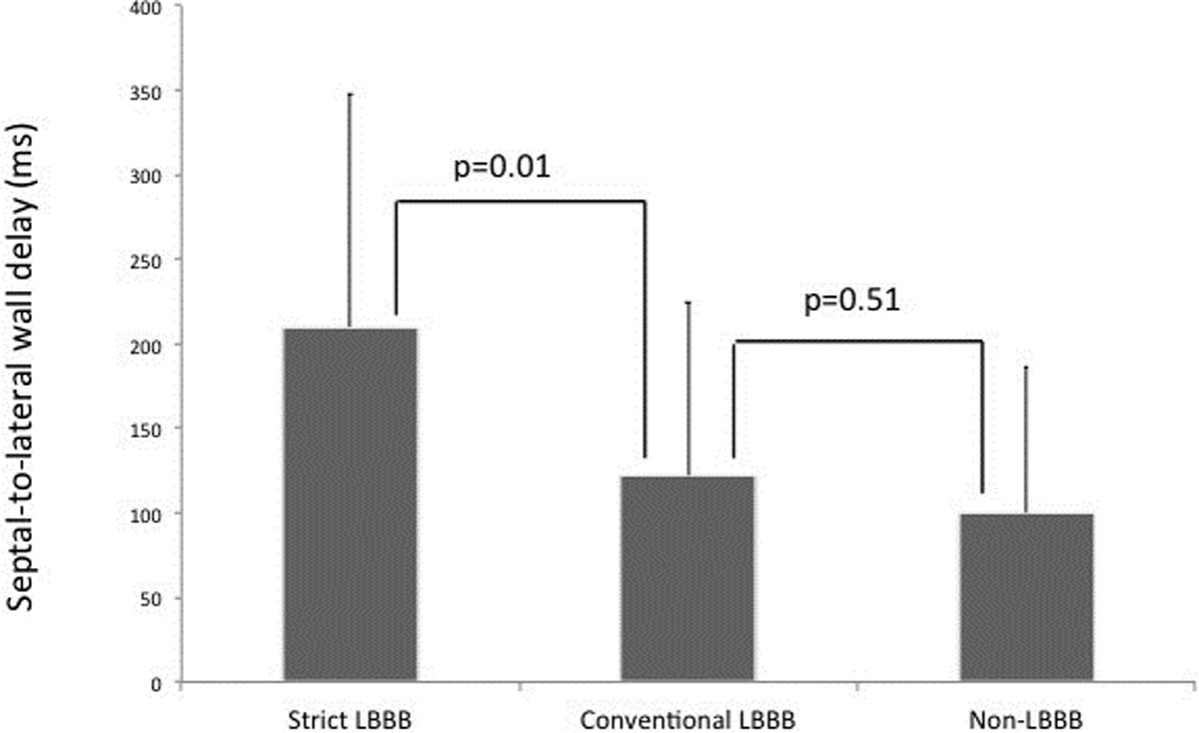
Reynolds Foundation and the FDA Critical Path Initiative. Septa-to-lateral wall delay for Strict LBBB, Conventional-only LBBB and Non-LBBB

## Conclusions

Strict LBBB ECG criteria identify patients with greater mechanical dyssynchrony compared to patients only meeting conventional-only LBBB criteria, while there was no significant difference between conventional-only and non-LBBB patients. The greater observed LV dyssynchrony may explain why strict-LBBB patients have better response to CRT.

## Funding

The study was supported by the National Heart, Lung, and Blood Institute, National Institutes of Health (HL103812 to KCW, HL91062 to GFT, and HL61912 to RGW), the DW

